# GABA through the Ages: Regulation of Cortical Function and Plasticity by Inhibitory Interneurons

**DOI:** 10.1155/2012/892784

**Published:** 2012-06-26

**Authors:** Konrad Lehmann, André Steinecke, Jürgen Bolz

**Affiliations:** Institut für Allgemeine Zoologie und Tierphysiologie, Universität Jena, 07743 Jena, Germany

## Abstract

Inhibitory interneurons comprise only about 20% of cortical neurons and thus constitute a clear minority compared to the vast number of excitatory projection neurons. They are, however, an influential minority with important roles in cortical maturation, function, and plasticity. In this paper, we will highlight the functional importance of cortical inhibition throughout brain development, starting with the embryonal formation of the cortex, proceeding by the regulation of sensory cortical plasticity in adulthood, and finishing with the GABA involvement in sensory information processing in old age.

## 1. Introduction

The functioning of the cerebral cortex depends critically on the precise balance between excitatory and inhibitory neurotransmitter systems. Excitation is mediated via glutamate by pyramidal neurons, the projection neurons of the cortex, and by a special class of local neurons in cortical layer IV, the spiny stellate cells. Inhibition is mediated via *γ*-aminobutyric acid (GABA) by cortical interneurons, which regulate the degree of glutamatergic excitation, filtering the input and regulate the output of projection neurons. GABAergic interneurons, the “nonpyramidal cells” of the cerebral cortex, take many different forms of dendritic and axonal arborization, which have been used for their morphological classification ever since their first description by Ramon *y* Cajal [1–5]. Moreover, interneurons also differ in their firing patterns, the neuropeptides they express, their calcium-binding protein content, and other molecular markers such as ion channels, receptors, and transporters. Based on the combination of structural, functional, and biochemical criteria, interneurons have been subdivided into many different subclasses and it is still a matter of hot debate among the experts of how many interneuron subtypes exist in the cortices of different species [6–8]. 

At the circuit level, interneurons control the flow of information and synchronization in the cerebral cortex. There are about five times more glutamatergic neurons than GABAergic neurons in the neocortex; this ratio is consistently observed across many mammalian species. This then suggests that the numerical balance of excitatory and inhibitory neurons may be important for normal brain function and behavior. Even though GABAergic interneurons comprise only a small fraction of the cells in the neocortex, disturbances in their development, and hence the delicate balance between excitation and inhibition, can lead to neurological or neuropsychiatric diseases such as epilepsy, autism, and schizophrenia (reviewed in [9–13]). These disorders often emerge during childhood and adolescence. However, as we will describe later in this paper, alterations in GABAergic interneuron can also occur in the adult and aging brain, with important repercussions for cortical function and plasticity.

## 2. Setting the Balance: Interneuron Migration into the Cortical Plate

**Figure 1:**
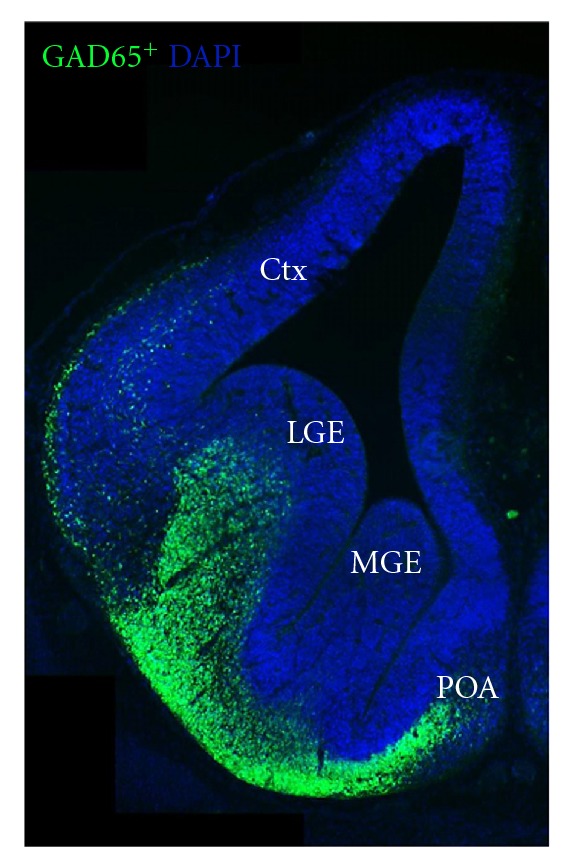
GABAergic interneurons are born in the basal telencephalon. Coronal cryosection of an E14 GAD65 EGFP mouse embryo. The MGE and the POA give rise to tangentially migrating cortical interneurons (green). GABAergic LGE-derived neurons migrate to the olfactory bulb, the striatum, and the lateral cortex. Ctx: cortex, MGE: medial ganglionic eminence, LGE: lateral ganglionic eminence, POA: preoptic area.

Cortical projection neurons, the excitatory pyramidal cells, arise in the ventricular zone (VZ) of the dorsal telencephalon and then migrate radially to form the laminated neocortex [14]. In contrast, GABAergic interneurons originate from the VZ of the ventral telencephalon from three regions: different interneuron subtypes are generated in the medial ganglionic eminence (MGE), the caudal ganglionic eminence (CGE), and the preoptic area (POA) (see Figure 1). For example, parvalbumine positive and somatostatin-positive interneurons arise mainly from the MGE, while most calretinin-positive interneurons are born in the CGE and the POA gives birth to vasoactive intestinal peptide and neuropeptide Y-positive interneurons. Interneurons originating from these regions then migrate tangentially in separate streams over long distances towards their cortical destinations [15–22].

The cellular and molecular mechanisms that regulate and guide interneuron migration out of the ganglionic eminences and POA into the neocortex are beginning to be described. Different groups of signaling molecules, including semaphorins and slits, act as repulsive cues for migrating interneurons [23, 24]. On the other hand, two isoforms of neuregulin act as short- and long-term attractants that demarcate the migratory route of cortical interneurons [25]. Another group of signaling molecules that is expressed widely in the basal telencephalon during interneuron migration is the ephrins and their receptors, the Eph receptor tyrosine kinases. As will be described later, recent findings from our lab provided direct evidence for distinct roles of Eph/ephrin interactions in the guidance of cortical interneuron migration.

The mammalian Eph/ephrin system consists of a family of receptor tyrosine kinases subdivided into 9 EphAs and 5 EphBs. A-type receptors bind to all A-type ephrins (ephrinA1–5), which are tethered to the cell membrane by a GPI anchor. B-type receptors bind to all B-type ephrins (ephrinB1–3), which have a transmembrane domain that is followed by a short cytoplasmatic region. An exception is EphA4, which can bind to both A-type and B-type ligands [26–28]. A distinctive feature of this signaling system is that an Eph receptor can also act as a ligand in the same manner that an ephrin ligand can act as a receptor. Ephrin binding induces Eph forward signaling; however, ephrins can also signal into the cell, which is called reverse signaling (for review, see [29–31]). Using a library of riboprobes for all members of the Eph/ephrin gene family, we systematically mapped with *in situ* hybridizations the complete set of these wiring molecules at different developmental stages. Our results revealed that many members of the Eph/ephrin system can be detected in the developing telencephalon and that they exhibit highly dynamic expression patterns [32, 33]. Based on the spatial and temporal expression patterns we could make some prediction about the potential roles of these wiring molecules in regulating the tangential migration of cortical interneurons. These hypotheses have then been tested with different bioassays *in vitro* and in diverse gene-targeted mouse lines directly *in vivo*.

**Figure 2:**
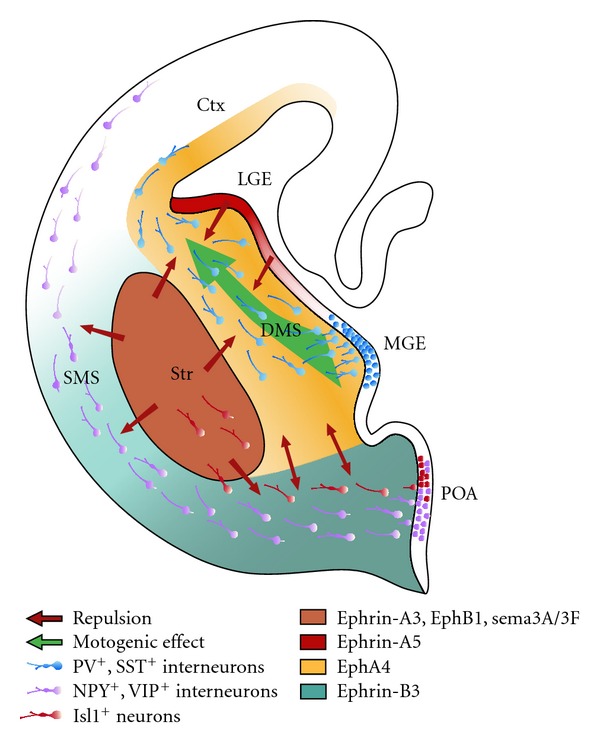
Different Eph/ephrin members act in concert to channel migrating MGE-derived neurons towards the cortex. Schematic drawing of a coronal brain slice from the right hemisphere; medial is right; dorsal is top. The MGE gives rise to parvalbumin-(PV-) and somatostatin-(SST-) positive interneurons. They are driven by EphA4 reverse signalling and guided by ephrin-A5, ephrin-A3, and Sema3A/3F forward signalling. The POA gives rise to neuropeptide Y (NPY), vasoactive intestinal protein (VIP) as well as islet 1 (Isl1) positive interneurons that are guided by EphB1/ephrin-B3 signalling preventing the POA-derived cortical interneurons from entering the striatum (Str). The same Eph/ephrin signalling allows the Isl1^+^ neurons to migrate towards the Str. The MGE gives rise to the deep migratory stream (DMS) and the POA provides neurons for the superficial migratory stream (SMS). Those streams are divided by a bidirectional EphA4 and ephrin-B3 signalling.

For example, we could demonstrate that ephrin-A3, which is expressed in the striatum, prevents migrating cortical interneurons from invading this inappropriate region [32]. We could also show that ephrin-A5 is expressed in the VZ of the ganglionic eminences, the dorsal boundary of the migratory route of MGE-derived interneurons, and that this molecule serves as an inhibitory border to channel these neurons into the subventricular zone [34]. Thus, as illustrated in Figure 2, the deep corridor of migrating cortical interneurons is at least in part defined by the concerted action of two different ephrin-A ligands, with ephrin-A5 flanking the dorsal portion and ephrin-A3 the ventral portion of this migratory stream.

**Figure 3:**
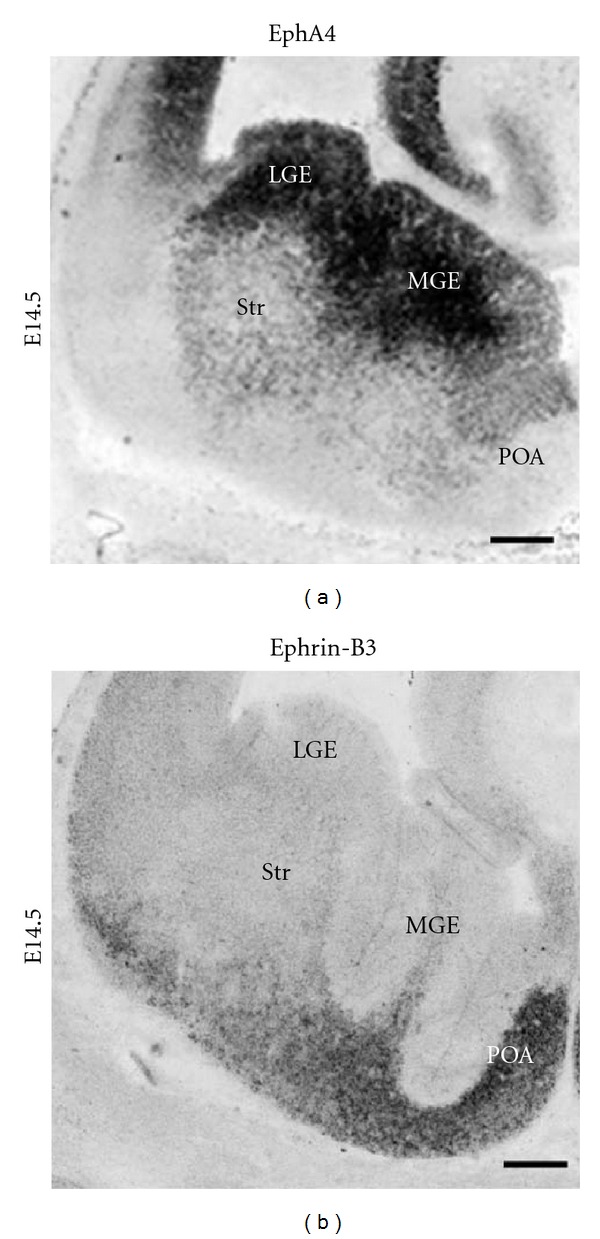
Complementary EphA4 and ephrin-B3* in situ* hybridization expression patterns. On embryonic day E14.5 EphA4 and ephrin-B3 are complementary expressed in the basal telencephalon of mouse brains. Based on these expression patterns several predictions about the functional role of these wiring molecules on interneuron migration could be made (see text for details). Scale bar: 100 *μ*m.

**Figure 4:**
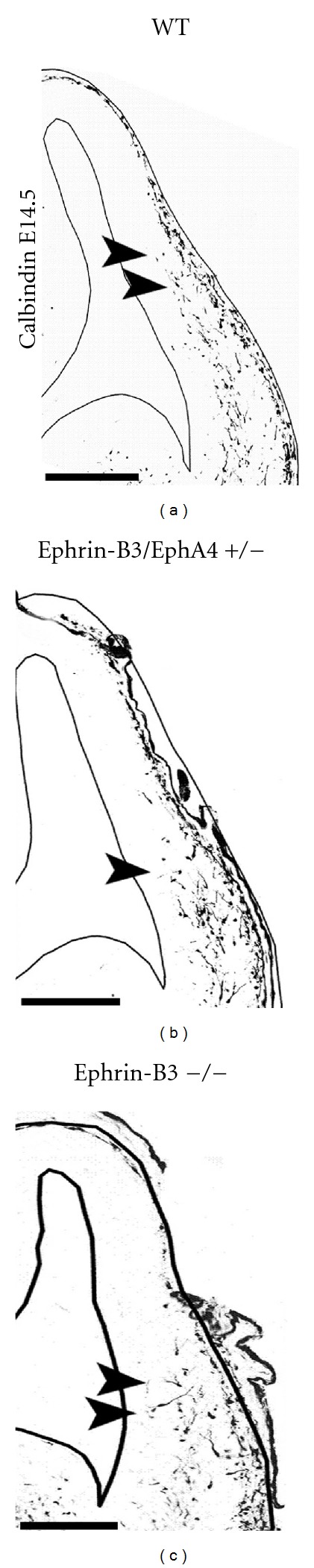
Alterations in the Eph/ephrin system cause a delayed migration of cortical interneurons *in vivo. *Analysing the calbindin-(CB-) positive interneurons in the cortex of E14.5 embryos revealed a delayed migration of interneurons in hetero- and homozygotic ephrin-B3/EphA4 knockout mice compared to the wild-type (WT) littermates. Arrow heads indicate the front of migrating interneurons. Scale bar: 100 *μ*m.

These repulsive effects are mediated by the EphA4 receptor, which is expressed by cortical interneurons [32, 34]. As already mentioned above, particular interneuron subtypes are generated in a temporally regulated manner in the MGE, CGE, and POA of the basal telencephalon. We could reveal that POA- and MGE-derived cortical interneurons migrate within spatially segregated corridors. Ephrin-B3, expressed in POA-derived interneurons traversing the superficial route, acts as a repellent signal for deeply migrating interneurons born in the MGE, which is mediated by EphA4 forward signaling. In contrast, EphA4 induces repulsive ephrin-B3 reverse signaling in interneurons generated in the POA, restricting this population to the superficial path (Figure 3). Perturbation of this bidirectional ephrin-B3/EphA4 signaling *in vitro *and in ephrin-B3/EphA4 double mutants *in vivo *leads to a partial intermingling of cells in these segregated migratory pathways and—as shown in Figure 4—to a delayed migration of calbindin-positive interneurons to the cortex. Thus cell contact-mediated bidirectional ephrin-B3/EphA4 signaling mediates the sorting of MGE- and POA-derived interneurons in the deep and superficial migratory stream [33].

We could also demonstrate that EphA4-induced reverse signaling has a motogenic effect of MGE-derived interneurons. In these experiments we first used different *in vitro* assays for cell migration and found that recombinant EphA4 stimulates the migratory speed of cortical interneurons. Thus, in addition to its established role in providing cell-contacted mediated repulsion, EphA4 can also tune the molecular machinery for neuronal migration. The ephrin ligands mediating EphA4 reverse signaling and the signal transduction cascade involved in this process are currently under investigation. However, in order to study the function of EphA4 on interneuron migration *in vivo*, we already examined cortical interneurons in an EphA4 knockout mouse line. We found that there was a delayed relocation of calbindin-positive interneurons into the cortex [35].

## 3. Disrupting Interneuron Migration by Disrupted-in-Schizophrenia 1

Disrupted-in-Schizophrenia 1 (DISC1) is a prominent susceptibility gene for major psychiatric disorders [36, 37]. The biological functions attributed to the DISC1 protein are complex and highly diverse. For example, previous work suggested that DISC1 plays an important role during neuronal proliferation, differentiation, neurite outgrowth, and synapse formation (reviewed in [38, 39]). There are also some studies that report that DISC1 is a necessary component for the correct positioning of radially migrating cortical pyramidal neurons [40, 41]. This prompted us to study the potential role of DISC1 for interneuron migration.

**Figure 5:**
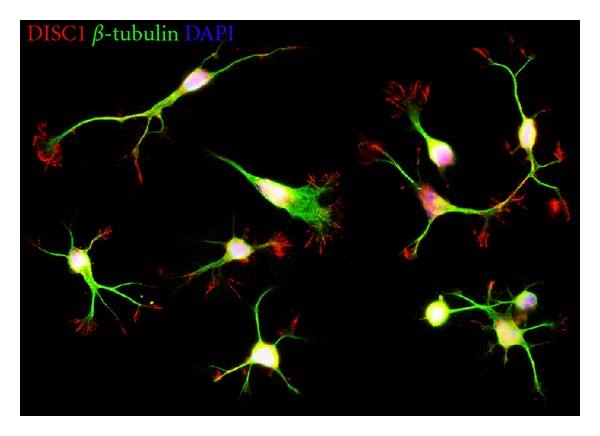
DISC1 immunocytochemistry on MGE-derived interneurons. Photomicrograph of MGE-derived neurons immunostained with DISC1 antibodies (red) and *β*-Tubulin antibodies (green) show a strong DISC1 signal in the processes of the interneurons.

**Figure 6:**
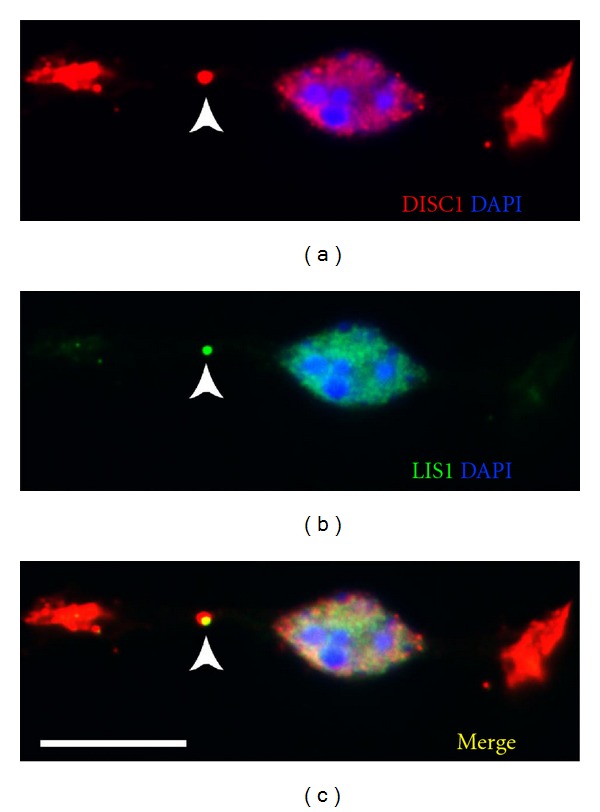
DISC1 and LIS1 immunocytochemistry on MGE-derived cells. Photomicrograph of an MGE-derived cell that was coimmunolabeled with a DISC1 antibody (a) and a LIS1 antibody (b). (c) represents the merged picture of (a) and (b). Note the precise overlap of DISC1 and LIS1 at the centrosome (yellow in c) pinpointed by the arrow heads. Scale bar: 10 *μ*m.

For this we first performed RT-PCR, *in situ* hybridization and immunostainings to verify that DISC1 is expressed in the MGE at the appropriate developmental stages. We also examined the subcellular distribution of DISC1. As illustrated in Figure 5, DISC1 is expressed in the tips of the leading processes. In addition, we also found DISC1 immunoreactivity at the rear of the nucleus, opposite to the leading process. A closer inspection revealed that DISC1 colocalizes with LIS1, previously described as a centrosomal protein [42] (Figure 6). Thus DISC1 is found in important strategic positions to control the molecular machinery involved in interneuron migration, for example, by interacting with cytoskeletal proteins tubulin and actin, motor proteins of the dynein and kinesin family, and regulatory proteins [40, 43]. 

To examine the functional role of DISC1 during interneuron migration, we performed *in utero *and *ex utero *electroporation to suppress DISC1 in the MGE *in vivo *and *in vitro*. Our results indicate that, after DISC1 knockdown, the proportion of tangentially migrating MGE neurons that reached their cortical target was reduced by 15%. In addition, there were profound alterations in the morphology of DISC1-deficient neurons, which exhibited longer and less branched leading processes than control cells [44].

These results indicate that DISC1 has an impact on the migratory behaviour of interneurons during early development that might lead to deficits in the number and/or composition of GABAergic neurons in the cortex. As mentioned in Section 1, dysfunctions of local GABAergic circuits have been often associated with the pathophysiology of schizophrenia [12]. Thus our findings support the notion that schizophrenia is a neurodevelopmental disease that may result from defects in interneuron integration [45].

## 4. Inhibitory Regulation of Sensory Cortical Plasticity

**Figure 7:**
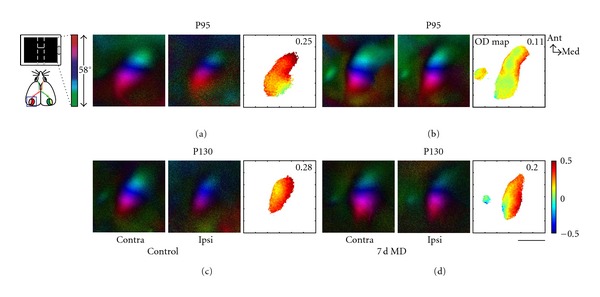
Adult ocular dominance plasticity ceases at postnatal day (P) 100. Retinotopic maps of the binocular visual cortex are shown. Elevation is colour coded according to the scheme on the left. Polar maps obtained by stimulation of the contralateral (contra) or ipsilateral (ipsi) eye are illustrated for four representative mice. Ocular dominance (OD) in the visual cortex is shown in the OD maps on the right, colour-coded according to the scheme on the right. In nondeprived control mice both before (a) and after (c) P100, the contralateral eye activates the cortex more strongly than the ipsilateral eye, which is reflected in warm-coloured OD maps. Seven days of monocular deprivation shift OD towards the ipsilateral eye in P95 animals ((b), colder colours of OD map), but have no such effect in the P130 animal (d). Scale: 1 mm.

Vulnerability for psychiatric diseases is not uniform throughout life, but is increased during certain stages of pre- and postnatal development [46, 47]. The factors that make neuronal subsystems especially open for environmental influences during well-defined, so-called critical periods have been the subject of much research in the neurosciences. As a typical example, the binocular visual cortex exhibits a well-studied critical period during which, under undisturbed conditions, the orientation preferences for stimuli seen through one versus the other eye are harmonised in visual cortical neurons [48]. It has long been known that temporary closure (i.e., monocular deprivation) of one eye during this critical period will shift the excitability of cortical neurons towards the open eye [49, 50]. This critical period for ocular dominance plasticity starts a few days after eye opening in mice, has a maximum at about postnatal day 28, and ends at around postnatal day 32, when short periods of deprivation have no detectable effect in the cortex [50]. Longer periods of deprivation, however, are still able to induce ocular dominance plasticity until postnatal day 100 (P100), but no longer (Figure 7, [51]).

The mechanisms regulating this period of enhanced plasticity have been the subject of intense research for many years. It has become obvious that the critical period is initiated by a shift in the cortical balance of excitation and inhibition [52, 53] (see [54] for review), a shift that is effected by the maturation of fast-spiking GABAergic interneurons, the so-called basket cells which are characterised by their expression of parvalbumin [55]. Thus, the start of the critical period is delayed in knockout mice lacking the GABA synthesising enzyme GAD65 and is triggered in these mice as soon as inhibition at GABA_A_ receptors is increased by the intracerebral infusion of diazepam [52]. Enhanced inhibition in early childhood prepones the start of the critical period [53, 56, 57]. On the other hand, several interventions have been shown in recent years to reinstate critical period-like plasticity in adult animals by reducing intracortical inhibition: treatment with the antidepressant fluoxetine allows for ocular dominance plasticity in adult rats [58]; the effect is accompanied by reduced cortical GABA levels and prevented by diazepam infusion. Additionally, fluoxetine treatment promotes the recovery from amblyopia of adult rats [58]. The same holds true for environmental enrichment, and again diazepam infusion averts the effect [59]. Indeed, directly attenuating GABA release by a GAD inhibitor reestablishes CP-like plasticity in adult rats [60].

Thus, it seems that GABAergic neurons single-handedly regulate visual cortical plasticity, and this impression is even enhanced by the recent observation that intracortical transplantation of embryonal cells from the medial ganglionic eminence (MGE), which are destined to become GABAergic cortical interneurons, induces a period of enhanced plasticity in mice beyond the critical period [61]. Interestingly, this effect is only present during a narrow time frame when the transplanted cells have a certain cellular age (33 to 35 day) corresponding to the age they would have had during the natural critical period in normal development. 

Surprising as this active time window may be, it is in line with a lot of research on the activity-dependent maturation of parvalbumin-containing interneurons in the visual cortex. Before and during the critical period for ocular dominance plasticity, the strength of cortical inhibition triples. This increase is prevented by dark rearing which also delays the critical period [62]. Further research specified that the number of perisomatic boutons around pyramidal cells in the visual cortex increases until postnatal day 28, which marks the peak of the critical period; again, visual deprivation prevented the increase [63]. These results imply that the maturation of basket cells is necessary for the start of the critical period for ocular dominance plasticity, and they show that this maturation is activity dependent. As mentioned above, transsynaptic transfer of the homeoprotein Otx2 from the retina, which is triggered by light perception, has been shown to induce the maturation of parvalbumin-containing interneurons and the start of the critical period [55]. A high concentration of polysialic acid (PSA), which traps Otx2 [64], has been shown to prevent the start of the critical period, such that premature removal of PSA leads to an earlier maturation of parvalbumin-expressing cells and a preponed critical period [65]. Recent work has elucidated to a large degree the cell-autonomous mechanisms that promote the developmental synapse formation in basket cells: a knockdown of the GABA-synthesizing enzyme GAD67 leads to deficits in axon branching and perisomatic synapse formation, whereas overexpression of GAD67 speeds up these processes [66], (see [67] for review). Thus, inhibitory innervation patterns are regulated by the cell's own activity, which in turn depends on GABA synthesis. An interesting recent twist in this story is the finding that the complete blockade of GABA synthesis in single cells does not, as one would expect, shrink axonal arbors drastically, but contrariwise increases their density and complexity [68]. The authors of that study suggest a model according to which basket cells make tentative contacts with many potential postsynaptic targets, which are pruned or stabilized by synaptic activity. While little activity is sufficient to remove, but not to stabilize connections, complete blockade allows for neither and therefore keeps axonal complexity and synapse number high [68]. In summary, visual stimulation induces the maturation of parvalbumin-containing basket cells, which is internally regulated by GABAergic activity, and shifts the excitation-inhibition balance of visual cortical neurons such that critical period plasticity becomes possible.

While it seems established that GABAergic inhibition controls the level of cortical plasticity, it is much less clear in how far GABAergic neurons are involved in the expression of plastic changes. Even after the drastic reduction of visual cortical inhibition just about the level that would evoke seizures, the ocular dominance shift evoked by monocular deprivation can be observed, and complete silencing of intracortical connections by muscimol confirms that the effective changes are expressed at thalamocortical synapses [69]. Indeed, ocular dominance plasticity is dependent on Hebbian plasticity at NMDA receptors [70–72]. Do inhibitory interneurons, then, participate in synaptic plasticity at all?

Several studies have tried to answer this question. Using calcium imaging, one recent study showed that GABAergic neurons are more binocular, that is, less dominated by one eye, in normal mice, but show a similar shift toward the open eye after monocular deprivation during the critical period [73]. If monocular deprivation was performed after the critical period, the ocular dominance shift of GABAergic neurons was even stronger than that of excitatory neurons. Another study, however, provided somewhat conflicting results: *In vivo* intracellular recording from pyramidal cells and fast-spiking interneurons showed that while excitatory cells have a normal bias towards the contralateral eye which they rapidly lose after a short monocular deprivation, fast-spiking interneurons were unbiased at the outset, showed a paradoxical shift towards the closed eye after short deprivation, and only shifted on to an ipsilateral bias after longer deprivation periods [74]. The discrepancies may arise from differences in anaesthesia and method or simply from the fact that the latter study focused on fast-spiking, parvalbumin-containing interneurons which play a central role in regulating the critical period for ocular dominance plasticity, whereas the Kameyama study did not distinguish among GABAergic neuronal subtypes. Both studies agree, however, that intracortical inhibition changes in response to monocular deprivation, and in adult plasticity, this change may even have a stronger influence on network function than the relatively small change in excitatory transmission [73].

In another primary sensory area, the somatosensory cortex, the role of GABAergic interneurons in the response to sensory deprivation has been firmly established in recent years (see [75] for review). If a single row of whiskers was removed in mice starting on postnatal day 7, the number of parvalbumin-positive interneurons was significantly reduced in the cortical barrels representing that row, whereas it was increased in adjacent barrels [76]. This loss of parvalbumin expression went along with a lower number of perisomatic synaptic varicosities and weaker inhibitory transmission, an effect that required experience-dependent release of BDNF [77]. Further research confirmed the reduction in parvalbumin expression and showed that, upon whisker trimming, fast-spiking interneurons in the barrel cortex, but not other kinds of nonpyramidal cells, become less excitable, while their excitatory thalamocortical input is reduced [78]. Interestingly, these findings are somewhat at variance with earlier research demonstrating that in rats in which a row of whiskers was plucked between postnatal days 1 and 60, the number of GABAergic synapses on dendritic spines, but not on somata, was strongly reduced in the deprived barrels [79] (see [80] for review). A potential approach to reconcile these conflicting results might be that whisker trimming at a very early age delays the maturation of cortical inhibition in a similar way as dark rearing does in the visual cortex. Here, this kind of deprivation also results in reduced parvalbumin expression [81]. As parvalbumin-positive interneurons mature quickly before the onset of the critical period for ocular dominance plasticity in the visual cortex [63, 82], so they do in the somatosensory cortex between postnatal days 10 and 30 [83]. It is conceivable that only a longer period of deprivation (two months in [79]) allows for the full adaptation to changed input.

Although such issues still need to be clarified, it appears that the mechanisms regulating sensory plasticity are similar in different cortical areas (see [84] for review), involving the expression of parvalbumin and the formation of perisomatic synapses by basket interneurons in both the somatosensory and the visual cortex. In the auditory cortex, too, similar processes seem to control critical period plasticity [85, 86]. This uniformity of function across the neocortex holds a promise for our ability to understand cortical plasticity, but a lot of work still remains to be done to understand the cellular and network mechanisms by which plasticity is enabled.

## 5. Age-Related Decline of Inhibition and Signal Processing

**Figure 8:**
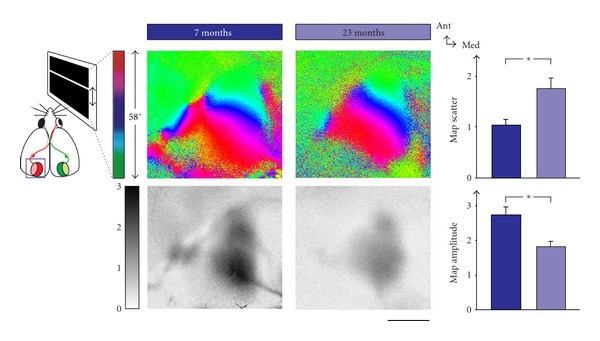
Visual cortical maps deteriorate in old age. Retinotopic phase maps (colour-coded according to scheme on the top left) and corresponding activity maps (coded according to grey scale on the left) are shown for mice of seven and 23 months. Map scatter increases in old mice, while the amplitude decreases. Scale bar is 1 mm and applies to all panels showing maps.

Perceptual sensitivity declines in old age. In aged humans, visual acuity and contrast sensitivity decline in an accelerated fashion [87–89]. In order to test in how far mice might serve as an animal model of age-related vision loss in humans, we have just shown that, in pigmented mice, too, visual acuity and contrast sensitivity deteriorate, starting at approx. 18 months of age, and the progressive loss parallels that which is seen in humans [90]. While age-related degradations in the sensory organs certainly impair access to the environment, there has been a long-standing notion that central nervous changes may also contribute to the decline in visual or somatosensory function [91, 92]. Indeed, we could show in the same study that visual cortical activity, as measured by optical imaging of intrinsic signals, and cortex-dependent behavioural plasticity were strongly reduced in old mice (Figure 8, [90]). 

A precise connection between cellular changes and visual function loss in old animals has been achieved in macaques. In these animals, orientation tuning of visual cortical neurons is reduced to the point of being scarcely detectable [93, 94]. Electrophoretic application of GABA or the the GABA agonist muscimol to the recorded neurons, however, restored orientation selectivity similar to that seen in young animals, whereas the GABA receptor antagonist bicuculline abolished orientation tuning in visual cortical neurons of young monkeys [94]. A similar degradation of visual cortical function, that is, decreased orientation sensitivity, higher spontaneous activity, and lower signal-to-noise ratio, was observed in aged cats and, partly, in rats [95, 96]. Looking for the reason of this decrease in intracortical inhibition, Hua and colleagues [97] found the density and proportion of GABA-immunopositive neurons in the cat visual cortex to be decreased by about half in old compared to young animals, whereas there was no change in the number of excitatory neurons.

Does this loss of inhibitory interneurons affect all GABAergic subtypes equally? In humans and dogs it appears that basket cells characterised by parvalbumin are remarkably resilient to old age [98, 99]. This fits to the most recent finding that the activity of parvalbumin-containing interneurons has almost no effect on the orientation tuning of visual cortical pyramidal cells [100], whereas a genetically induced loss of dendrite-targeting interneurons leads to impaired orientation tuning in the mouse visual cortex [101]. The density of calbindin-immunoreactive interneurons, in contrast, is diminished in almost all of the aged human brain, but significantly so only in a few areas, among them the primary visual cortex [98]. The density of calretinin-positive neurons is also affected by age, but mostly in temporal areas. Somewhat conflicting results were found in the somatosensory and motor cortices of rats, where the density of both parvalbumin- and somatostatin-positive interneurons was found to be decreased in old animals [102]. It is as yet unclear whether the discrepancy concerning parvalbumin-positive cells is due to differences in brain region, strain, or species. It appears certain, however, that some kinds of GABAergic interneurons are lost during ageing and that this loss may be the reason for the functional degradation found in old animals. This knowledge may provide a basis for future therapeutic interventions.

## 6. Conclusions

Despite being only a minority of all cortical neurons, inhibitory interneurons have a key role in modulating cortical function and plasticity, and even subtle impairments to the integrity of these cells can lead to severe neuronal and psychiatric disturbances. It is therefore crucial to understand the development of GABAergic interneurons, their integration into cortical circuits, and the factors necessary for their preservation. In consequence, it might become possible to treat neuronal disorders at their basic circuit level.
